# Milk-Sickness

**Published:** 1861

**Authors:** Isaac Mendenhall

**Affiliations:** Ashland, Ind.


### MILK-SICKNESS.

VALEDICTORY ADDRESS DELIVERED BEFORE THE NEW CASTLE MEDICAL SOCIETY.

BY ISAAC MENDENHALL, M.D., Ashland, Ind.

GENTLEMEN:—Soon after the organization of our society, several members seemed anxious that this question should be discussed. In order to bring the subject before you, your President appointed at different times a member to read an essay on the subject. They having failed, I thought that I would at least give it a passing notice, and thereby get the opinions of other members.

*History.*—As far as we know, the first notice of the disease was published in Cincinnati, 1809, and consisted of the observations of Dr. Barbee, a highly intelligent physician of Bourbon county, Ky., who, on a visit to the upper settlements on the great Miami river, first became acquainted with it.* Since that time our medical journals have presented a great many papers on this malady; but notwithstanding the ability with which some of them have been drawn up, much obscurity and doubt envelope the malady. Kentucky, liberal in her efforts to relieve the afflicted, several years ago offered a reward for the discovery of the remote cause or causes of the disease; but no one has yet appeared to claim the prize with success.

This disease has been known in the States of North Carolina, Virginia, Tennessee, Kentucky, Ohio, Indiana, and Illinois. I believe it has not yet appeared west of the Mississippi river. In these regions it has been known at different times and different places; but it appears to be more prevalent in summer and autumn, though no season is entirely exempt from its ravage.

I have called the disease *milk-sickness*, from which you will infer that it is a disease produced in man by the milk of the cow, that contains a "peculiar" poison. Prof. Slack terms it *ergodelateria*, and says it is caused by the cattle eating blasted grain. As yet, we have no proof that blasted grain produces

* According to the late Dr. Drake, it was known in North Carolina eighty years ago. *Vide Western Journal of Medical and Physical Sciences*, ix., 243, which I found after penning the above.

the disease. As yet, we are entirely *ignorant* in regard to the cause of the *malady* in the gramntvorous animals: man generally receives the poison by using the flesh, milk, butter, cheese, etc., of the gramnivorous animals that have received a charge of the poison. Dogs eating of the flesh get the slows, and are unable to attend to their masters' calling. Buzzards cannot fly after eating the poisoned flesh; and all animals that eat of the dead carcasses are more or less affected. The disease is known by the animal trembling on exertion. Whilst at rest, no manifestations of disease can be observed. In many instances, it is no uncommon occurrence for cattle to drop down dead after being drove some four or five miles, that present no symptoms of the disease prior to the travel. Immoderate exercise is not a prerequisite of the manifestation of the disease: they will tremble while in their ordinary occupation of grazing, and die without the heat of labor. Calves will eject their milk and fall down and die, whilst sucking; and hogs will eject their food. These are the common symptoms by which the disease is known in the animals, until a post-mortem examination reveals the following phenomena: The stomach and intestines are inflammed; the mucous coat is measurably destroyed; the entire coats are in some cases gangrenous; they are a dark, or, I might say of a black appearance. This black color is not a mere congestion; the coats are not firm, though they are not easily broken down. In the ventriculus, or paunch, and, indeed, throughout the whole alimentary canal, is found a substance conglomerated and resembling cemented sawdust, in the shape of balls, somewhat elongated, some of them perfectly adherent to the coats of the digestive tube, whilst others are loose.

So far as I am acquainted with the semeiology of the diseases of animals, and their morbid appearances after death, there is no disease analogous to the one under consideration; as stated before, there is nothing known in regard to the cause, up to this time. Cultivation of the soil is considered a sure preventive; we may therefore infer that it is of vegetable origin. The horse, mule, cow, goat, sheep, hog, dog, and buzzard, have all been known to have the disease.

Symptoms in man: All ages are susceptible to an attack, though children are not so apt to be taken down by its invasion as the adult. When the invalid is about to be taken down he feels weary, and trembles more or less on exertion, and experiences pain, numbness, and slight cramps of the extremities; at the same time he generally becomes costive, and under fatigue is likely to experience nausea; his appetite is not generally impaired at first, but has a feeling of depression and burning at the stomach; is irresolute, and is as much indisposed to mental as bodily effort. He may continue in this situation for some time and recover spontaneously, or with the aid of a mild cathartic; but more commonly, under the influence of an exciting cause, severe symptoms supervene: a full meal, indigestible food in the stomach, or exertion, is such an one. The invalid being subject to these, full vomiting comes on, with much epigastric distress. He throws up the contents of the stomach, and, continuing at short intervals to vomit or retch, brings up small quantities of acrid mucus, tinged in streaks or spots of an indigo color, but very seldom any bile. As the disease advances it becomes of a muddy, dirty appearance; the vomiting is so obstinate in many cases that not a particle of medicine or fluid can be retained for some time; there seems, indeed, to be an inverted peristaltic action of the stomach and bowels; during the intervals the patient lies on his back, and throws himself from side to side of the bed. There is an insatiable thirst: his urgent desire is water, to allay, as he confidently expects, the burning sensation of the stomach, which is a constant symptom; its soothing effects are but temporary; it is soon ejected, and he urgently calls for more. In the midst of these symptoms his bowels remain torpid, though in some cases diarrhœa is present. But little pain is complained of in the bowels, and they do not appear to swell, but the contrary, and seem retracted and reduced in volume; so much so that the pulsations of the abdominal aorta can be distinctly felt, and is found beating with unwarranted violence. The discharges from the bowels consist at first of the natural excrements contained in them. They soon change to a dirty soap-suds appearance, and very offensive to the olfactory organs; after the use of medicine, they look as near like jelly and green moss chewed and mixed together as anything I can compare it to. The breath is generally offensive, somewhat resembling the smell of ptyalism and chloroform. I heard some of the farmers say it resembled the odor evolved from the rattlesnake. I have frequently noticed the same fætor to a considerable extent in the breath of children affected with worms.

In the forming stages there is but little change in the appearance of the tongue, but it soon coats over with a light white fur; after a few days it inclines to change to a brown color, particularly the middle part; the tip and edges are then of a red and fiery color. The pulse is but little increased in frequency at first, most generally a little below the normal range of action; but as the disease advances, and places the patient in imminent jeopardy, the pulse increases in frequency. The respiration is with an occasional sigh, with a distressing sense of oppression in the chest. If the disease is not arrested, the irritation of the stomach increases; and if the discharges were not black before, they soon become so, and sometimes assume the coffee-ground color of the yellow fever. The breathing becomes more oppressive, with a sense of sinking of the chest; and an inability to supply the want is felt in the chest. The pulse is now small, frequent, and feeble, the countenance shrunken and anxious, the surface cool or cold, and cold extremities, whilst the trunk is generally hot, or warmer than natural. After several days, perhaps a week or more from the commencement, if the bowels have not been evacuated, they are apt to give way spontaneously; tympanitis takes place, and the patient has frequent griping pains, and frequent watery mucous discharges, tinged with blood. The tongue becomes dry, red, and fissured, the dorsum is covered with a dark fur, sordes collect about the teeth, soreness of the throat, with difficult deglutition, sometimes occur, and the patient lies on his bed with his legs drawn up. These symptoms may continue a few hours, or perhaps a few days, when stupor comes on, the pulse ceases to be felt at the wrist, and death takes place, preceded by profound coma. A favorable termination of the disease is marked by a gradual diminution of all the characteristic symptoms, until they disappear, though the convalescence is sometimes very tedious; and a liability to relapses remain for a long time, which may be called into action by an over-meal, fatigue, excessive exertion, or exposure to either excessive heat or vicissitudes of temperature.

But little is known in regard to the pathology. Public opinion is so averse to post-mortem examinations that it is very rare that a physician has the opportunity to make one. Dr. Graff says, in a woman who died of the disease he found inflammation of the meninges, and congestion with softening of the substances of the brain. The stomach was slightly contracted, and the mucous membrane was reddened in patches; there were a few ounces of bloody scrum in the peritoneal cavity, the liver deeply engorged, and darker than in health;—and the gall-bladder was distended with bile. Dr. William Trafton, of Evansville, Ind., is stated by Dr. Byford to have found inflammation of the mucous membrane of the stomach, rigid contraction of the pyloric orifice, a dry and hard condition of the feculent matter in the bowels, and a total absence of intestinal gases. Dr. Dickey, in a case that died with all the symptoms of the disease, found, on opening the abdomen, the peritoneum was inflamed and gangrenous, the major curvature of the stomach was injected of a bright pink color;—the lesser curvature and orifices were pale—the contents resembled a chalk mixture intermingled with globules of oil.—This was oleum ricini, that had been taken during his illness. The mucous coat was much destroyed, and deprived of epithelium, there being an appearance of having suppurated throughout its whole surface of the duodenum and stomach. Brunner's gland was enlarged and red, but no signs of ulceration. Passing on to the jejunum, we found still more evidence of inflammatory action in this as well as the ilium; the mucous coat was easily separated by the finger nail, and the external surface of the latter presented flakes of white and green.—Peyer's patches were decidedly ulcerated. The contents of the cœcum were much the same as the ilium; the coats were found to be all affected, of a muddy, dirty appearance, some parts inclining to a green color. The large intestines contained much sulphuretted hydrogen. Colon in some parts rather pale, in other gangrenous. There was ramolissement of the liver. The border of the right lobe dark and indurated, left lobe mottled. The lobus spigelii and quadratus quite normal. The gall-bladder was much distended with a thick, tenacious fluid. Mesenteric glands motley. The organs situated in the thorax were healthy. By comparing the morbid appearances as here presented with those of the animal, we will find that the mucous coats of the stomach and intestines suffer most. Although the evidence here is not gleaned from many cases, yet those from the animal are founded on numerous dissections.

*Prognosis.*—This is a very grave disease. The physician should not promise too much: we cannot tell what amount of poison we have to deal with. When pressed for an opinion, give it cautiously. If the patient has a tolerably good constitution, and called early, it will generally end well.

*Diagnosis.*—The prominent diagnostic characters of milk sickness are: Violent and incessant vomiting, generally constipation, absence of bile in the matters discharged, a retracted state of the abdomen, and a hardened condition of the same. The heart manifests a convulsive action, and excessive palpitation of the abdominal aorta, throughout the course of the disease, distressing burning and sinking of the stomach and bowels. Great mental discomfort; an excessive and peculiar odor of the breath; and with these the evidence of encephalic or abdominal inflammation, and in bad cases a depraved state of the blood, and groat nervous prostration.

*Treatment.*—Venesection has been but seldom employed in this disease, and should not be recommended only where the brain and its membranes appear to be affected. Cupping the abdomen and scrobiculus cordis may be used to an advantage. Cathartics are the remedies universally employed. We need not state the individual experience of physicians in regard to their use; both they and the people make the indication of opening the bowels the indication of cure, and affirm that when the bowels are freely moved the patient rapidly recovers. In the forming stages, catharsis is generally easy to be accomplished; after vomiting has commenced, the difficulty is greatly increased. We consider that when the bowels are freely moved in a severe case, our labors are only commenced. Our plan is this: Give from thirty to sixty grains of calomel;—and if the bowels are not moved within four hours, give olcum ricini, or an infusion of senna and epsom salts. If these do not have the desired effect within three or four hours more, I use enemas composed of jalap, aloes, olcum ricini, and warm water; use them every hour until the bowels are moved freely. The calomel I give in sugar and water; if it is ejected, I repeat every half hour until I get a dose to stay down. If the irritability of the stomach should continue very long, I use sinapisms or epispastics; in some cases, a cloth dipped in cold water and applied to the scrobiculus cordis has a very sanative effect. The above course is persisted in and repeated every twenty-four hours, until the green, mossy appearance of the discharges is changed to a more biliary character. Experience has convinced me that it is perfectly useless to give calomel in alterative doses, in this disease; by giving it in cathartic doses, it will lie in the system long enough to have all the alterative effect that is needed.

I am aware that there are some physicians opposed to the use of calomel in this disease, for what good reason I do not know. I have tried to get along without it, but found that I was losing valuable time, not being able to find any other cathartic that would remain long enough to do any good. We find the vitiated secretions and excretions collecting in the alimentary canal daily, until the poison is expelled from the system; hence it is that it is so necessary to keep up a continued regular action in the bowels, at the same time keeping the secreting organs aroused to their normal action, or as near as possible; at the same time we give stimulants during the whole course of disease, if there is an indication for their use. We endeavor to keep the pulse up to 75 or 80 beats to the minute; and if they flag much below that point, we give stimulants in appropriate doses, and repeat them according to the emergencies of the case; on the other hand, if the pulse is above ninety beats to the minute, we withhold them, especially if they have much force about them, until it is brought down to the normal standard, or a little below that point. If we stimulate too freely while the pulse is up to 100 or more, we most assuredly assist the disorganizing process already set up in the digestive tube or brain.

Some physicians pour the spirits into them without measure and without caution, thinking that if they can get liquor enough in them that their cases will get well; all such practice, to say the least of it, is reprehensible, and fraught with danger. It is true, some will get well under such practice,—some will get well if nothing is done; so it is with the most of other diseases, when the indisposition is slight. There is also a variety of opinions advanced in regard to opiates; we give them, occasionally, to allay the irritability of the stomach, and to procure rest.

Cold water is interdicted in this disease, and for what good reason I do not know; by allowing a portion of spirits to be added to each draught, we may allow a moderate portion given. An effervescing draught, composed of bicarb. soda and tart. acid, with the addition of a small portion of spirits, may be used, which is very grateful to the patient, and has a soothing effect in allaying the intense burning in the stomach. The spirits we have used are New England rum, wine, French brandy, alcohol. If we find that our patient appears to be sinking, with cold extremities, we have found epispastics applied to them to have a very good effect. If there is much cerebral difficulty, we apply cold water freely to the head.—Dr. Toland has also subjected his patients to the cold dash, and, he affirms, with apparent advantage. The diet must be mild, and easy of digestion: panada, toast, broths, soup, chicken-broth, etc. During convalescence the action of the bowels must be kept up, and hearty meals avoided; but above all, the patient must refrain from active exercise. When the recovery is slow, tonics are useful, especially chalybeates.

The above is a brief summary of my views of the disease. Although I have given rather an outline course of treatment, there are often many other indications to be fulfilled, which the enlightened and energetic practitioner will readily perceive. We might have went on and discussed the "mineral theory," the "vegetable theory," etc., but it would not have proved anything different than what has already been advanced, and already expressed, in regard to the different cathartics recommended by different members of the profession. A great variety have been used; some use croton oil. Dr. Bruller gave sulphate of magnesia and whiskey, and considered nothing else necessary. Dr. J. W. Crooks, who avers he has not lost a single case for 15 years, used alcoholic drinks, morphine and blisters, to allay the vomiting, and states that if compelled to limit his choice to a single remedy, he would select whiskey. He thinks that alcohol neutralizes the poison, and in its tura neutralizes the alcohol, as it is almost impossible to produce intoxication. He prefers, for the cathartic effect, sulphate of magnesia and calcined magnesia. As stated before, we prefer calomel for a cathartic in milk-sickness, before any other and all other cathartics.

There is no disease that requires more prompt and close attention than the one under consideration. The physician should put in as near all his time at the bed-side as possible, so as to be on the alert whenever any change takes place. It is a disease that will not bear dallying with very long. If any of you wish to investigate any further, I would refer you to Wood's Practice of Medicine, fifth edition, p. 460; Dickey on Milk-Sickness, Western Lancet, vol. xiii., p. 390; Drake on Milk-Sickness, Western Journal of Medicine and Surgery, vol. iii., p. 161, which have been consulted in writing this essay.—*Lancet and Observer.*

